# Epigenetic Modifications in Head and Neck Cancer

**DOI:** 10.1007/s10528-019-09941-1

**Published:** 2019-11-11

**Authors:** Jadwiga Gaździcka, Karolina Gołąbek, Joanna Katarzyna Strzelczyk, Zofia Ostrowska

**Affiliations:** Department of Medical and Molecular Biology, Faculty of Medical Sciences in Zabrze, Medical University of Silesia, Jordana 19 Str., 41-808 Zabrze, Katowice, Poland

**Keywords:** HNSCC, Squamous cell carcinoma, Epigenetics, Methylation, MicroRNA, Histone modification

## Abstract

Head and neck squamous cell carcinoma (HNSCC) is the sixth most common human malignancy in the world, with high mortality and poor prognosis for patients. Among the risk factors are tobacco and alcohol intake, human papilloma virus, and also genetic and epigenetic modifications. Many studies show that epigenetic events play an important role in HNSCC development and progression, including DNA methylation, chromatin remodeling, histone posttranslational covalent modifications, and effects of non-coding RNA. Epigenetic modifications may influence silencing of tumor suppressor genes by promoter hypermethylation, regulate transcription by microRNAs and changes in chromatin structure, or induce genome instability through hypomethylation. Moreover, getting to better understand aberrant patterns of methylation may provide biomarkers for early detection and diagnosis, while knowledge about target genes of microRNAs may improve the therapy of HNSCC and extend overall survival. The aim of this review is to present recent studies which demonstrate the role of epigenetic regulation in the development of HNSCC.

## Introduction

Epigenetic modifications can be defined as heritable, reversible changes in gene expression which do not result from a change in the sequence of DNA bases (Momparler 2003; Teodoridis et al. 2004; Castilho et al. 2017). Therefore, epigenetic mechanisms change the phenotype without interference in DNA sequences (Arantes et al. 2014). Epigenetic processes include DNA methylation, histone posttranslational covalent modifications, changes in chromatin structure, and effects of non-coding RNAs (Arantes et al. 2014; Castilho et al. 2017). An epigenetic pattern may be modulated by external factors such as diet, alcohol, tobacco, toxins, or pharmaceutical treatment (Ghantous et al. 2018). Epigenetic mechanisms are associated with carcinogenesis of numerous cancers (Park et al. 2011; Osorio and Castillo 2016) and play an important role in the development of head and neck squamous cell carcinoma (HNSCC).

HNSCC is placed at sixth of the most frequent human malignancies and belongs to the most aggressive cancers. Worldwide, more than half a million new cases are diagnosed per year and nearly 50% of them have a less than 5-year survival rate (Leemans et al. 2011; Ganci et al. 2012; Magić et al. 2013). Cancer cells spread to the oral cavity, larynx, naso-, hypo-, and oro-pharynx. Environmental and genetic factors influence the development of HNSCC and the main lifestyle risk factors include Human Papilloma Virus (HPV) infection, age, diet, tobacco use, and alcohol intake (Demokan and Dalay 2011; Magić et al. 2013; Koffler et al. 2014). External factors like alcohol abuse and tobacco influence epigenetic patterns in some types of HNSCC like oral cancer (Ghantous et al. 2018).

In this review, we summarize the main epigenetic modifications associated with HNSCC.

## Methylation of DNA

**Fig. 1:**
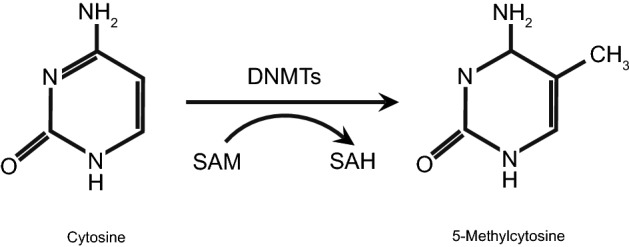
DNA methylation. DNMTs add a methyl group (CH_3_) to the carbon in the 5 position of cytosine, converting it to 5-methylcytosine. The donor of the methyl group is S-adenosylmethionine (SAM), which is converted to S-adenosylhomocysteine (SAH) (updated from Luczak and Jagodziński 2006; Osorio and Castillo 2016)

DNA methylation, the covalent addition of a methyl group (CH_3_) to carbon in the 5 position of cytosine in the sequence 5′-CG-3′, is one of the most common epigenetic mechanisms (Fig. 1) (Momparler 2003; Luczak and Jagodziński 2006; Gopisetty et al. 2006; Magić et al. 2013). The targets of methylation may be in any type of DNA sequence such as intergenic DNA, genes, or non-coding repetitive sequences (Reyngold and Chan 2018). However, in the genome CpG dinucleotides are arranged asymmetrically; in normal cells single CpGs are highly methylated, while CpG islands (CGIs), 0.5–4 kb regions of DNA which content 60–70% of CG dinucleotides, are usually unmethylated. Approximately 50% of genes contain CGIs in their promoter regions (Luczak and Jagodziński 2006; Magić et al. 2013; Reyngold and Chan 2018) and methylation mostly occurs in the promoter region or the first exon sequence (Luczak and Jagodziński 2006; Gopisetty et al. 2006; Arantes et al. 2014). Methylation is catalyzed by members of the family of DNA methyltransferases (DNMTs) composed of DNMT1, DNMT2, DNMT3A, and DNMT3B. DNMT1 is a maintenance enzyme responsible for methylation during replication, while de novo methylation is catalyzed by DNMT3A and DNMT3B (Luczak and Jagodziński 2006; Arantes et al. 2014). The DNMT family enzymes use S-adenosylmethionine (SAM) as a methyl donor, which is converted to S-adenosylhomocysteine (SAH) (Luczak and Jagodziński 2006; Osorio and Castillo 2016).

Methylation is an important process to regulate gene expression, especially in silenced genes located on the inactivated X chromosome in females and in genomic imprinting (Luczak and Jagodziński 2006; Park et al. 2011; Arantes et al. 2014; Reyngold and Chan 2018). Aberrant patterns of methylation have been reported in various cancers, and two different patterns of methylation are present. The first is genome-wide hypomethylation, and the second is hypermethylation of CGIs located in the promoter region of genes. Global hypomethylation is connected with chromosomal instability and gene activation, while increased methylation in promoter regions concerns mostly tumor suppressor genes and in consequence reduces their expressions (Gopisetty et al. 2006; Arantes et al. 2014).

### Hypomethylation

Global DNA hypomethylation in repeat sequences, transposons, gene deserts or CpG dinucleotides located in introns may influence genome instability (Ehrlich 2009; Hatziapostolou and Iliopoulos 2011). In the other hand, it may stimulate activation of oncogenes or latent viruses (Magić et al. 2013; Castilho et al. 2017). Hypomethylation of promoters of genes or retrotransposons has been documented in studies of HNSCC. Lower methylation in retrotransposon elements, like long interspersed elements (LINEs) or short interspersed elements (SINEs), influences carcinogenesis through genome destabilization. In normal mammalian cells LINE sequences have a high methylation status, while during cancer development they are hypomethylated, which contributes to activating transcription of sequences which influence genome instability and as a result may facilitate carcinogenesis (Luczak and Jagodziński 2006; Reyngold and Chan 2018). LINE-1 (long interspersed nucleotide element-1) has decreased methylation in various cancer cells compared to normal cells, and meta-analysis shows that this hypomethylation is associated with advanced cancer (Kitkumthorn and Mutirangura 2011). In addition, another meta-analysis, based on 20 studies concerning methylation of repeated sequences such as LINE, Alu, and Sat-α as prognostic markers for various cancers, suggests that the level of global DNA hypomethylation is connected with a dismal prognosis (Li et al. 2014b). Several studies show hypomethylation of LINEs or SINEs in head and neck cancer (Richards et al. 2009; Subbalekha et al. 2009; Chaisaingmongkol et al. 2012). Hypomethylation of Alu, one of the SINEs, was confirmed by Puttipanyalears et al. (2013), who reported that in oral cancer among the Asian population the Alu methylation decreased with advanced stages of cancer. Foy et al. (2015) showed that in patients with premalignant oral lesions, LINE sequences are hypomethylated and associated with increased risk for oral carcinogenesis. Furthermore, LINE-1 sequences are hypomethylated in oral squamous cell carcinoma (OSCC), but independently of the tumor's clinical stage and location (Subbalekha et al. 2009), as well as in oropharyngeal squamous cell carcinoma (OPSCC) (Furlan et al. 2017). Interestingly, OPSCC patients with LINE-1 hypomethylation have a 3.5-fold higher risk of early relapse compared to cases with higher methylation (Furlan et al. 2017). On the other hand, Smith et al. (2007) showed that LINE-1 hypomethylation occurs in 67% of HNSCC cases examined and that global hypomethylation is connected with an advanced stage of the tumor using ANOVA, although this correlation was not significant by multivariate analysis. Arayataweegool et al. (2019) utilized cocultures of HNSCC cell lines and peripheral blood mononuclear cells (PBMCs) to measure the methylation level of LINE-1 in PBMCs, and found that this level is significantly downregulated in coculture with cancer cells due to factors secreted by HNSCCs, an effect which could be used for HNSCC diagnostics. Kitkumthorn et al. (2012) measured the methylation level of LINE-1 and Alu sequences in lymph node (LN) metastases of HNSCC patients and confirmed their lower methylation in HNSCC samples with metastasis; however, only hypomethylation of LINE-1 was statistically significant and furthermore the decreases of methylation levels were not associated with the stage and grade of tumors. It has been also reported that global hypomethylation is characteristic for patients with tongue squamous cell carcinoma (TSCC) and, interestingly, is connected with female gender. In addition, Chen et al. (2016a) observed associations between decreased methylation and poor survival for TSCC patients, predominantly for female, older patients with a stage I or II AJCC (American Joint Committee on Cancer) cancer without lymph node involvement and with postoperative radiotherapy. On the other hand, Morandi et al. (2017) observed no signs of hypomethylation in OSCC. Hypomethylation in promoter regions of genes has been shown in several studies of HNSCC (Table 1).

**Table 1:** Selected genes which are hypomethylated in HNSSC

Gene	Cancer	Observed connections with hypomethylation	References
*WSIP1*	OSCC	Higher expression of WSIP1 protein characteristic for patients with lymph node metastasis	Clausen et al. (2016)
*CSPG4*	HNSCC	Worse clinical outcome Overexpression of mRNA and protein	Warta et al. (2014)
*PD-L1*	HNSCC	Overexpression of PD-L1 protein	Franzen et al. (2018)
*PD-L2*	HNSCC	Upregulation of PD-L2 mRNA expression	Franzen et al. (2018)
*IL6*	OSCC	Upregulation of gene expression	Basu et al. (2017)
*PTPN22*	OSCC	Upregulation of gene expression	Basu et al. (2017)
*RUNX1*	OSCC	Upregulation of gene expression	Basu et al. (2017)
*CD28*	OSCC	Upregulation of gene expression	Basu et al. (2017)
*CD22*	OSCC	No data related to gene expression	Basu et al. (2017)
*CD80*	OSCC	Upregulation of gene expression	Basu et al. (2017)
*TLR1*	OSCC	Upregulation of gene expression	Basu et al. (2017)
*TNFa*	OSCC	Upregulation of gene expression	Basu et al. (2017)
*APEX2*	HNSCC	Decreased mRNA expression in tumor	Chaisaingmongkol et al. (2012)
*TREX2*	HNSCC	No data related to gene expression	Chaisaingmongkol et al. (2012)
*MSH4*	HNSCC	Decreased mRNA expression in tumor	Chaisaingmongkol et al. (2012)
*MIR296*	OSCC	No date related to gene expression	Morandi et al. (2017)
*TERT*	OSCC	No date related to gene expression	Morandi et al. (2017)
*GP1BB*	OSCC	No date related to gene expression	Morandi et al. (2017)

### Hypermethylation

**Fig. 2:**
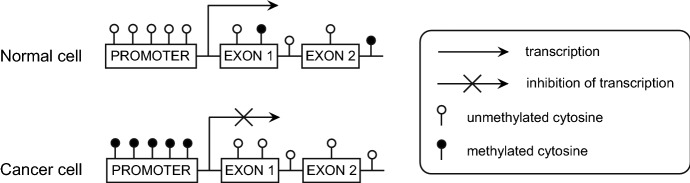
Methylation of promoter region of genes in normal and cancer cells (updated from Hatziapostolou and Iliopoulos 2011; Reyngold and Chan 2018)

In normal cells, CGIs are poorly methylated in transcriptionally active genes, while a high level of methylation in promoters of genes is characteristic for epigenomes of cancer cells (Castilho et al. 2017; Reyngold and Chan 2018). This hypermethylation of CGIs may lead to transcriptional silencing of tumor suppressor genes and in consequences promote malignant transformation (Fig. 2) (Herman and Baylin 2003; Magić et al. 2013). Hypermethylation of promoter regions in head and neck cancer has been shown in many studies, and below we summarize recent studies about *p16, PTEN*, *DAPK*, *MGMT*, *ECAD* and *RASSF1* genes which are frequently analyzed in HNSCC. Genes which are less common hypermethylated in HNSCC are presented in Table 2.

**Table 2:** Selected genes which are hypermethylated in HNSCC

Gene	Cancer	Observed connections with hypermethylation	References
*SALL3*	HNSCC	No data related to gene expression	Chaisaingmongkol et al. (2012)
*FANCB*	HNSCC	No data related to gene expression	Chaisaingmongkol et al. (2012)
*NEIL1*	HNSCC	Lower gene expression	Chaisaingmongkol et al. (2012)
*AGTR1*	OSCC	Association with OSCC development	Foy et al. (2015)
*FOXI2*	OSCC	Association with OSCC development	Foy et al. (2015)
*PENK*	OSCC	Association with OSCC development	Foy et al. (2015)
*LXN*	OSCC	Downregulation of gene expression	Basu et al. (2017)
*HLA-DPB1*	OSCC	Upregulation of gene expression	Basu et al. (2017)
*ZNF577*	OSCC	Downregulation of gene expression	Basu et al. (2017)
*ZNF154*	OSCC	Downregulation of gene expression	Basu et al. (2017)
*CTDSP1*	OSCC	Downregulation of gene expression	Basu et al. (2017)
*ZSCAN31*	OSCC	Not significant downregulation of gene expression	Basu et al. (2017)
*LDLRAD4*	OSCC	Not significant downregulation of gene expression	Basu et al. (2017)
*NDN*	HNSCC	Association with overall survival time	Virani et al. (2015)
*CD1A*	HNSCC	Association with overall survival time	Virani et al. (2015)
*GRIM-19*	HNSCC	Significant independent risk factor for HNSCC, and Not significant downregulation of gene expression	Zhang et al. (2015b)
*PTEN*	NPC	Downregulation of gene expression	Li et al. (2014a)
	OSCC	No data related to gene expression	Sushma et al. (2016)
*PAX1*	HNSCC	Downregulation of gene expression	Guerrero-Preston et al. (2014);
	OSCC	No data related to gene expression	Morandi et al. (2017)
*PAX5*	HNSCC	Downregulation of gene expression	Guerrero-Preston et al. (2014)
*ZIC4*	HNSCC	No data related to gene expression	Guerrero-Preston et al. (2014)
*PLCB1*	HNSCC	No data related to gene expression	Guerrero-Preston et al. (2014)
*LRPPRC*	TSCC	No data related to gene expression	Bhat et al. (2017)
*RAB6C*	TSCC	Downregulation of gene expression	Bhat et al. (2017)
*ZNF471*	TSCC	Downregulation of gene expression	Bhat et al. (2017)
*MINT1*	HNSCC	No data related to gene expression	Choudhury and Ghosh (2015)
*MINT2*	HNSCC	No data related to gene expression	Choudhury and Ghosh (2015)
*MINT31*	HNSCC	No data related to gene expression	Choudhury and Ghosh (2015)
*TFPI2*	OSCC	Downregulation of protein expression	Kim et al. (2019)
*SOX17*	OSCC	Downregulation of protein expression Association with overall survival time	Kim et al. (2019)
*GATA4*	OSCC	Downregulation of protein expression Association with overall survival time	Kim et al. (2019)
*ESRRG*	LSCC	Association with overall survival time	Shen et al. (2019)
*HOXA9*	HNSCC	Possible association with progression and Metastasis of HNSCC	Zhou et al. (2019)
*ZNF671*	HNSCC	Downregulation of gene and protein expression Association with overall survival time	Zhang et al. (2019)
*RHCG*	HNSCC	Downregulation of gene expression Association with overall survival time	Xu et al. (2019)
*SALL1*	HNSCC	Downregulation of gene expression Association with risk of disease recurrence	Misawa et al. (2018b)
*CEACAM6*	LSCC	Downregulation of gene expression	Bednarek et al. (2018)
*PCDH17*	LSCC	Downregulation of gene expression	Byzia et al. (2018)
*CPEB4*	HNSC	Downregulation of gene and protein expression	Zeng et al. (2018)
*CLDN11*	LSCC	Associated with lymph node metastasis, advanced clinical stage, and higher T classification	Shen et al. (2017)
*SLIT2*	HNSC	Associated with tumor location	Alsofyani et al. (2017)
*KL*	HNSC	Associated with high grade tumor	Alsofyani et al. (2017)
*SFRP1*	HNSCC	Association with poor survival for patients with moderately differentiated tumor (grade 2)	Alsofyani et al. (2017)
*PTPRD*	LSCC	Downregulation of gene expression	Szaumkessel et al. (2017)

The gene *p16* (*CDKN2A*) is a known tumor suppressor gene, which inhibits cyclin-dependent kinases and cell cycle progression (Magić et al. 2013; Padhi et al. 2017). Hypermethylation of its promoter is a common finding in HNSCC studies and meta-analyses (Sanchez-Cespedes et al. 2000; Don et al. 2014; Choudhury and Ghosh 2015; Sushma et al. 2016; Dvojakovska et al. 2018; Alsofyani et al. 2017; Veeramachaneni et al. 2019). Allameh et al. (2018) reported a higher methylation level of its promoter in OSCC patients compared to a control group, and hypermethylation was associated with lower expression of that gene in tumor samples. A meta-analysis comparing 67 case control studies confirms the higher methylation of the *p16* promoter region in HNSCC than in normal controls, and shows that the methylation level increases progressively from the control group to patients with premalignant lesions and then to HNSCC patients, respectively. Hypermethylation was associated with male gender as well as with LN metastasis. Methylated *CDKN2A* may therefore be a useful marker in diagnosis and prognosis for head and neck cancer (Zhou et al. 2018).

Another hypermethylated tumor suppressor gene in HNSCC is phosphatase and tensin homolog (*PTEN*), which negatively regulates Akt signaling and in consequence decreases cell proliferation (Sushma et al. 2016). Several studies reported hypermethylation in the promoter of *PTEN* in oral cancer (Alyasiri et al. 2013; Sushma et al. 2016), and nasopharyngeal cancer (Li et al. 2014a). Moreover, in OSCC, this increased methylation is associated with well-differentiated tumors and with age of under 50 years among an Indian population; no correlation was found between methylation and gender (Alyasiri et al. 2013). Increased methylation in the *PTEN* promoter in NPC tissues and NPC cell lines is connected with down-regulation of *PTEN* (Li et al. 2014a) and lower expression of *PTEN* mRNA in OSCC-derived cell lines (Tanzawa et al. 2008).

Death-associated protein kinase (*DAPK*), a tumor suppressor gene, is also hypermethylated in head and neck cancer (Sanchez-Cespedes et al. 2000; Choudhury and Ghosh 2015), and hypermethylation is positively correlated with LN metastases and with stages III and IV of HNSCC (Sanchez-Cespedes et al. 2000), as well as inversely correlated with lower expression of *DAPK* in tongue cancer (Bhat et al. 2017). A meta-analysis of eighteen studies confirmed that methylation of the *DAPK* promoter is over fourfold higher in HNSCC patients compared to healthy controls (Cai et al. 2017), while another meta-analysis confirmed *DAPK* promoter hypermethylation among OSCC patients (Don et al. 2014) as well as an association with a higher risk of nasopharyngeal carcinoma (Zhang et al. 2018a). The *DAPK* promoter is also more highly methylated in OSCC samples compared to matched surgical margins, and interestingly is associated with LN metastasis and older age of HNSCC patients (Strzelczyk et al. 2019).

The gene *MGMT* (O6-methylguanine-DNA methyltransferase) is related to DNA repair, and increased methylation in its promoter has been reported in HNSCC (Koutsimpelas et al. 2012; Chaisaingmongkol et al. 2012; Dvojakovska et al. 2018). In a meta-analysis based on 20 studies, the promoter of *MGMT* was hypermethylated in HNSCC compared to healthy controls, suggesting a connection between higher methylation and an increased risk of head and neck cancer (Cai et al. 2016). Meta-analysis of OSCC cases also confirmed higher methylation in this promoter (Don et al. 2014) and in addition, the increased methylation was connected with a lower level of MGMT protein (Koutsimpelas et al. 2012). Onerci Celebi et al. (2016) utilized a pyrosequencing technique to assay methylation level, and showed that hypermethylation of *MGMT* promoter is frequent in laryngeal cancer; however, without association with clinicopathological features of patients such as age, tumor stage or differentiation, and disease-free survival. Moreover, hypermethylation was found in HNSCC tumors compared to the surgical margins (Strzelczyk et al. 2018).

E-cadherin (*CDH1*, *ECAD*) is another tumor suppressor gene related to cell adhesion, and is frequently hypermethylated in HNSCC cases (Choudhury and Ghosh 2015; Strzelczyk et al. 2018). A meta-analysis based on 13 studies revealed that increased methylation of the promoter of *CDH1* is associated with oral cancer risk (Wen et al. 2018), and it has been suggested that this hypermethylation is associated with lower expression of E-cadherin protein in OSCC patients (Pannone et al. 2014). In contrast, Domingos et al. (2017) showed no differences in the methylation level of *CDH1* promoter between groups of patients with potentially malignant oral lesions, OSCC, and healthy controls; moreover, in most samples the *CDH1* gene promoter was unmethylated and in the OSCC group the level of methylation was not associated with clinicopathological features.

*RASSF1* (Ras association domain-containing protein 1) is a tumor suppressor gene which plays an important role in cell cycle control, apoptosis, and cellular adhesion, and its inactivation is associated with development of many cancers (Donninger et al. 2007). In head and neck cancer, hypermethylation of *RASSF1* promoter is a frequent event (Choudhury and Ghosh 2015) and meta-analysis shows that it is significantly associated with these cancers. It has been suggested that aberrant methylation of *RASSF1A* may be a useful biomarker for HNSCC (Meng et al. 2016). Also, a recent meta-analysis of OSCC confirmed that promoter hypermethylation of *RASSF1A* is connected with oral cancer risk (Wen et al. 2018) although, Koutsimpelas et al. (2012) observed hypermethylation of *RASSF1A* in only 13% of tumor samples examined.

### HPV Status and Level of DNA Methylation

Differences in methylation level occur between HPV(+) and HPV(-) HNSCC cases. In addition, HPV status influences aberrant methylation patterns in head and neck cancer independently of other external risk factors like smoking or alcohol (Degli Esposti et al. 2017). Several studies reported that HPV infection may be connected with hyper- or hypomethylation of genes, which are presented in Table 3.

**Table 3:** The level of methylation connected with HPV status

Gene	HPV status	Methylation	References
*CDH18*	+	↑	Degli Esposti et al. (2017)
*CTNND2*	+	↑	Degli Esposti et al. (2017)
*ELMO1*	+	↑	Degli Esposti et al. (2017)
*CDH8*	+	↑	Degli Esposti et al. (2017)
*CRMP1*	+	↑	Degli Esposti et al. (2017)
*PCDH10*	+	↑	Degli Esposti et al. (2017)
*MSX2*	+	↑	Degli Esposti et al. (2017)
*SYN2*	+	↑	Degli Esposti et al. (2017)
*PCDHB11*	+	↑	Degli Esposti et al. (2017)
*HTR1E*	+	↑	Degli Esposti et al. (2017)
*CCNA1*	+	↑	Colacino et al. (2013) and Virani et al. (2015)
*GRB7*	+	↑	Colacino et al. (2013)
*CDH11*	+	↑	Colacino et al. (2013)
*RUNX1T1*	+	↑	Colacino et al. (2013)
*SYBL1*	+	↑	Colacino et al. (2013)
*TUSC3*	+	↑	Colacino et al. (2013)
*MINT31*	+	↑	Choudhury and Ghosh (2015)
*NDN*	+	↑	Virani et al. (2015)
*CD1A*	+	↑	Virani et al. (2015)
*DCC*	+	↑	Virani et al. (2015)
*CADM1*	+	↑	van Kempen et al. (2014)
*TIMP3*	+	↑	van Kempen et al. (2014)
*ADORA2*	+	↑	Vogt et al. (2018)
*NCAN*	+	↓	Degli Esposti et al. (2017)
*NRXN1*	+	↓	Degli Esposti et al. (2017)
*COL19A1*	+	↓	Degli Esposti et al. (2017)
*SYCP2*	+	↓	Degli Esposti et al. (2017)
*RPA2*	+	↓	Degli Esposti et al. (2017)
*SMC1B*	+	↓	Degli Esposti et al. (2017)
*SPDEF*	+	↓	Colacino et al. (2013)
*STAT5A*	+	↓	Colacino et al. (2013)
*MGMT*	+	↓	Colacino et al. (2013)
*ESR2*	+	↓	Colacino et al. (2013)
*JAK3*	+	↓	Colacino et al. (2013)
*HSD17B12*	+	↓	Colacino et al. (2013)
*p16*	+	↓	Virani et al. (2015)
	+	↑	Choudhury and Ghosh (2015)
*RASSF1*	+	↓	Colacino et al. (2013)
	+	↑	Choudhury and Ghosh (2015)
*NT5E*	+	↓	Vogt et al. (2018)
*CHFR*	−	↑	van Kempen et al. (2014)
*PAX1*	−	↑	Guerrero-Preston et al. (2014)
*PAX5*	−	↑	Guerrero-Preston et al. (2014)
*CDH13*	±	↑	van Kempen et al. (2014)
*RARB*	±	↑	van Kempen et al. (2014)
*DAPK*	±	↑	van Kempen et al. (2014)
	+	↑	Choudhury and Ghosh (2015)
*LINE1 seguences*	+	↑	Furlan et al. (2017)
	*-*	↓	Richards et al. (2009)

“+” HPV-positive; “−” HPV-negative; “↑” hypermethylation; “↓” hypomethylation

### Smoking and Drinking Abuse and Their Potential Influence on DNA Methylation

Exposure to smoke and alcohol influences methylation level (Ghantous et al. 2018). Methylation linked with exposure to smoke or alcohol is presented in Table 4.

**Table 4:** Methylation linked with exposure to smoke or alcohol

Gene	Smoke	Alcohol	Methylation	References
*NDN*	+	No data	↓	Virani et al. (2015)
*CD1A*	+	No data	↓	Virani et al. (2015)
*DCC*	+	No data	↓	Virani et al. (2015)
*PAX1*	+	No data	↑	Guerrero-Preston et al. (2014)
*PAX5*	±	No data	↑	Guerrero-Preston et al. (2014)
*DAPK*	+	No data	↑	Arantes et al. (2015)
	No correlation	+	↑	Cai et al. (2017)
*CADM1*	−	−	↑	van Kempen et al. (2014)
*TIMP3*	−	No correlation	↑	van Kempen et al. (2014)
*RASSF1*	No correlation	No correlation	↑	Wen et al. (2018)
*p16*	+	No data	↑	Allameh et al. (2018)
	No correlation	No correlation	↑	Sushma et al. (2016)
*PTEN*	No correlation	No correlation	↑	Sushma et al. (2016)
*Alu seguences*	+	No data	↓	Puttipanyalears et al. (2013)
*Line1 sequences*	No correlation	No correlation	↓	Smith et al. (2007)
	No correlation	No correlation	↓	Subbalekha et al. (2009)

“+” used; “−” not used; “↑” hypermethylation; “↓” hypomethylation

### Methylation Enzymes

A level of enzymes associated with methylation and demethylation processes and their activity may influence epigenetic regulation. Below we present the recent studies on DNMTs and TET enzymes and their connections with HNSCC.

#### DNMT

The level and activity of DNMTs may contribute to HNSCC development. *DNMT3b* is upregulated in esophageal squamous cell carcinoma, and associated with hypermethylation of multiple tumor-associated genes such as *DAPK*, *p16*, or *CDH1* (Li et al. 2011). The mRNA expression of DNA methyltransferases (DNMT1, DNMT3a and DNMT3b) was upregulated also in OSCC. Moreover, overexpression of *DNMT1* was an independent marker of poor clinical outcome and relapse-free survival of OSCC patients (Supic et al. 2017). Another study also confirmed upregulation of DNMT3a in OSCC, in connection with low expression of Klotho, the anti-aging gene (Adhikari et al. 2017). Chen et al. (2016b) revealed that in invasive subclone HNSCC cell lines, DNMT3b was upregulated, while E-cadherin was downregulated, suggesting that DNMT3B may be involved in induction of epithelial–mesenchymal transition (EMT). Moreover, miR-29b mimic leads to a decrease of DNMT3b expression and inhibits EMT (Chen et al. 2016b). DNMTs expression may be associated with the expression of other epigenetic factors. Mochizuki et al. (2018) observed that overexpression of EZH2, member of Polycomb protein, is positively correlated with the upregulation of *DNMT3a* but not associated with *DNMT3b* in HNSCC. The level of enzyme may be also modulated by a dietary component such as folate (diet-derived methyl donor). Methyl donor depletion leads to increased expression of *DNMT3a* in HPV(+) HNSCC cell line, while *DNMT1* and *DNMT3a* expressions are either not altered or not significantly higher, respectively (Hearnden et al. 2018). DNMTs may be a potential target for enhancement of HNSCC chemotherapy by the use of inhibitors of DNMTs and reversal of genes methylation (Suzuki et al. 2009). It has been reported that DNMT1 was a target of miR-148a-3p in LSCC. It was found that the overexpression of miR-148a-3p downregulated DNMT1 expression, which led to upregulation of RUNX3, tumor suppressor, through decreasing its methylation (Jili et al. 2016).

#### TET

Ten-eleven translocation (TET) family of enzymes are pivotal factors of epigenetic regulation machinery through demethylation process. This family of 5-mC hydroxylases is composed of TET-1, TET-2, and TET-3 proteins, which catalyze the conversion of 5-methylcytosine to 5-hydroxymethylcytosine (Tahiliani et al. 2009). TET proteins are large enzymes of 180–230-kDa (Rasmussen and Helin 2016) and have a C-terminal catalytic domain with TET-1 and TET-3 containing also N-terminal CXXC zinc finger domain (Zhao and Chen 2013). TET proteins are involved in many important processes during mammalian development such as embryonic development, but also may influence tumorigenesis (Tan and Shi 2012). The lower expression of TET proteins occurs in malignant and solid tumors (Rasmussen and Helin 2016). During tumorigenesis, the TET activity is reduced by tumor hypoxia. Hypoxia influences increased promoter methylation and leads to decreased activity of TET enzymes in many tumors, also in HNSCC (Thienpont et al. 2016). Also, methyl donor depletion may influence expression of *TET-1*, what was confirmed by Hearnden et al. (2018). They showed that reduced methyl donor was associated with increased expression of *TET-1* in HPV-positive HNSCC cell line.

The aberrantly methylated TET enzymes in HNSCC patients were studied by Misawa et al. (2018a), who reported lower expression of *TET-1* and *TET-3* in HNSCC, while methylation level of these genes was higher in cancer cells, suggesting that downregulation of *TET-1* and *TET-3* must have been associated with their promoter methylation. Moreover, the multivariate analysis revealed that *TET-3* methylation in OSCC and oropharyngeal cancer was connected with poor survival of HNSCC patients (Misawa et al. 2018a).

Decreased expression of *TET-1* gene occurs also in laryngeal squamous cell carcinoma (LSCC) and is connected with a lower level of 5-hmC, suggesting that the level of 5-hmC is strongly correlated with the level of *TET-1* and may be a poor prognostic factor of LSCC patients in an early stage of cancer (Zhang et al. 2016). However, Zhang et al. (2016) did not find any significant differences in the expression of *TET-2* and *TET-3* between LSCCs and normal tissues. On the other hand, downregulation of TET-2 was correlated with a lower level of 5-hmC in OSCC patients (Jäwert et al. 2013). Wang et al. (2017) observed that the expression of 5-hmC was significantly reduced in oral cancer and the expression of *TET-2* was significantly lower in OSCC patients, which may be contributing to cancer development. Moreover, increased level of 5-hmC was correlated with decreased overall survival, suggesting its usability as a prognostic factor for OSCC (Wang et al. 2017). TET enzyme may influence a response to chemotherapy in HNSCC. Song et al. (2019) observed that TET-2 promoted 5-hmC formation after the administration of chemotherapeutic agents like doxorubicin. Moreover, PML (promyelocytic leukemia) recruited TET-2 to regulate DNA modification during chemotherapy of HNSCC, and as a result impaired cell proliferation. Furthermore, higher levels of TET and PML were associated with better overall survival of HNSCC patients (Song et al. 2018). Wang et al. (2018b) showed that a decreased expression of TET-1 in OSCC may lead to increased promoter methylation of MGMT, and enhanced the sensitivity of OSCC stem cells to chemotherapeutics like cisplatin.

## Histone Modifications

Histone modifications play a key role in regulating chromatin structure and DNA transcriptional activity, and aberrations in histone modification are associated with cancer (Bannister and Kouzarides 2011).

**Fig. 3:**
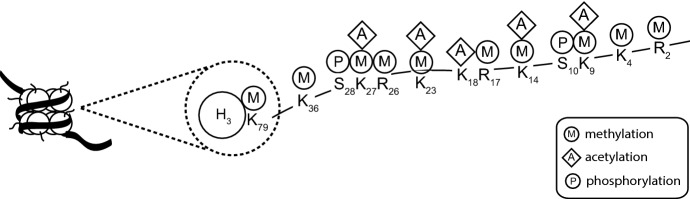
Posttranslational modifications of histones (updated from Bannister and Kouzarides 2011; Osorio and Castillo 2016)

A nucleosome, the primary unit of chromatin, is composed of four histone proteins (two copies of H2A, H2B, H3, and H4) which make a histone octamer, and 147 base pairs of DNA wrapped around it (Hatziapostolou and Iliopoulos 2011; Osorio and Castillo 2016). Histones are basic proteins which consist of a globular C-terminal domain and N-terminal tails. The tails undergo many different posttranslational modifications (PTMs) including acetylation, methylation, phosphorylation, ubiquitination, and ADP-ribosylation (Park et al. 2011; Hatziapostolou and Iliopoulos 2011) which modulate interactions between DNA and the histone octamer and in consequence the accessibility of the DNA (Bowman and Poirier 2015). PTMs are carried out by enzymes that add or remove the chemical group on the amino acids arginine, serine, or lysine (Fig. 3) (Hatziapostolou and Iliopoulos 2011; Bowman and Poirier 2015; Castilho et al. 2017).

### Histone Acetylation

Acetylation and deacetylation influence the conformation of nucleosomes and are catalyzed by histone acetyltransferases (HATs) and histone deacetylases (HDACs), respectively. Lysine acetylation results in relaxation of the chromatin structure, facilitating gene transcription (Momparler 2003; Castilho et al. 2017), while deacetylation silences genes by decreasing the accessibility of DNA to transcription factors (Momparler 2003). Deregulation of histone acetylation may cause increased transcription of various genes, and in consequence can lead to malignant transformation (Mancuso et al. 2009; Webber et al. 2017). Giudice et al. (2013) showed that in HNSCC cells, chromatin hypoacetylation occurs, which is evidenced by low level of histone H3 acetylated on lysine 9 (H3K9) in comparison to normal oral keratinocytes. In HNSCC patients, the level (expression) of histone H4 acetylated on lysine 16 (H4K16ac) is connected with early clinical stages of cancer, whereas histone H3 acetylated on lysine 9 (H3K9ac) is connected not only with the early clinical stages but also with increasing levels of differentiation and absence of lymph nodes (Noguchi et al. 2013). OSCC patients have hypoacetylated H3K9ac, and in addition this modification of chromatin condensation is connected with lower survival rate (Webber et al. 2017). Moreover, histone H3 acetylated on lysine 27 (H3K27ac) at the promoter of lncRNA PLAC2 leads to upregulation of PLAC2, and in results influences on OSCC progression via activating Wnt/β-catenin signaling pathway (Chen et al. 2019). A connection between tumor progression and histone acetylation was also found by Chen et al. (2013c), who showed that high expression of H3K18ac and a low level of H3K4a were associated with an advanced stage and a T status of oral cancer, while H3K4as additionally was associated with nodal invasion and poor survival. The level of expression of histone deacetylases has an influence on tumor progression; for instance, the overexpression of HDAC9 stimulates the development of OSCC by alterations of the cell cycle, cell proliferation, and apoptosis (Rastogi et al. 2016). These studies show that in oral cancer cases, the overexpression of HDAC2 is frequent and univariate analysis shows that higher HDAC2 expression is associated with shorter overall survival, suggesting that its level can be a useful prognostic marker for patients with oral cancer (Chang et al. 2009). Furthermore, in OSCC, increased expressions of mRNA and HDAC6 protein are detected. Interestingly, HDAC6 expression is associated with tumor aggressiveness (Sakuma et al. 2006). Almeida et al. (2014) found that HDAC inhibitors effectively protect against cisplatin resistance caused by NFκB signaling, which affects tumor resistance by histone deacetylation. Moreover, chemical inhibition of histone deacetylase classes I and II impairs HNSCC proliferation and decreases the fraction of cancer stem cells (Giudice et al. 2013; Castilho et al. 2017).

### Histone Methylation

Methylation of lysine, histidine, and arginine in histones is involved in changes of the chromatin structure and gene regulation but without altering the charge of histones. The methyl group is added to amino acid residues by histone methylases (HMT) while histone demethylases reverse this process. Lysine may occur in the mono-, di-, or tri-methylated form (Bannister and Kouzarides 2011; Castilho et al. 2017). The epigenetic effects of methylation depend on the location where the methyl group is added. Histone H3 has a few different lysine sites for methylation like K4, K9, K27, K36, or K79. An open chromatin structure results from methylation of H3K4, in contrast to methylation of H3K9 that causes the condensation of chromatin (Lachner and Jenuwein 2002; Momparler 2003; Le et al. 2014). Furthermore, histone methylation is a marker for both transcriptionally active (H3K4me3, H3K79me3 or H3K36me3) or silenced (H3K9me2, H3K9me3 or H3K27me3) genes (Bedi et al. 2014; Castilho et al. 2017). In OSCCC, a high level of H3K27me3 is associated with tumor progression (advanced T and N status, and stage of tumor), and also with disease-free survival as well as cancer-specific survival (Chen et al. 2013c). Furthermore, the level of H3K4me3 is decreased, whereas that of H3K4me2 is higher in OSCCC cases (Mancuso et al. 2009).

Histone modifications are also associated with the Polycomb protein complex which plays a key role in chromatin remodeling and transcription regulation (Sauvageau and Sauvageau 2010). One member of the Polycomb group is EZH2 (enhancer of zeste homologue 2) which regulates gene silencing by methylation at H3K27, whose expression is upregulated in OSCCC cell lines in comparison to cells from dysplasia or normal mucosae. In addition, overexpression is associated with clinical stage and tumor size and negatively correlated with the histological differentiation, leading Kidani et al. (2009) to suggest that the overexpression of that methyltransferase may be used as a prognostic marker for OSCC patients. On the other hand, EZH2 is overexpressed in HNSCC cell lines although this is not associated with an aberrant H3K27me3 status. Gannon et al. (2013) reported that inhibition of EZH2 may decrease the methylation level of H3K27 and in consequences may stimulate expression of differentiation genes in differentiation-refractory HNSCC cell lines. Additionally, higher expression of EZH2 mRNA was confirmed in HNSCC patients and is correlated with overexpression of DNMT3A, as well as with stage of cancer and recurrence, which suggests a role of EZH2 in tumor progression (Mochizuki et al. 2018). In OSCC, Chen et al. (2013b) found overexpression of methyltransferases for H3K9 and H3K27 (SUV39H1 and EZH2, respectively), which has consequences for prognostics; methyltransferase for H3K9 is associated with advanced tumor stage, while higher expression of EZH2 is positively correlated with LN metastasis. In TSCC cell lines and TSCC patient samples, mRNA for another member of the Polycomb complex, Bmi1 (B lymphoma Mo-MLV insertion region 1 homolog), is upregulated and associated with shorter survival, suggesting the possible use of Bmi1 expression as a prognostic marker (Li et al. 2014c).

### Histone Phosphorylation

Phosphorylation by adding a phosphate group from ATP is a modification occurring primarily on threonine, serine, and tyrosine located in N-terminal histone tails and is regulated by kinases and phosphatases. As a consequence, the histones have a lower positive charge which may influence chromatin organization (Bannister and Kouzarides 2011). The threonine protein kinase of H3S10 (ARK2) is overexpressed in oral cancer cases in the Taiwanese population and this upregulation in nuclei is connected with poor survival, while the cytosolic overexpression is correlated with the T status and stage of cancer. ARK2 may therefore be useful as a prognostic biomarker (Chen et al. 2013b). Furthermore, the overexpression of ARK2 in HNSCC patients was confirmed by Qi et al. (2007), who reported that higher expression of that enzyme is correlated with histological differentiation, cell proliferation, and metastasis in oral cancer, which indicates a role in OSCC progression.

### Histone Sumoylation

Sumoylation is a modification similar to ubiquitination in which three enzymes, E1, E2, and E3, add molecules of the small ubiquitin-like modifier (SUMO) to histone lysines (Bannister and Kouzarides 2011). There are also sumo-specific proteases (SENPs) which can reverse sumoylation, seven of which are known in humans (Ding et al. 2008). One of these is SNEP5, whose expression level has been reported to be higher in oral cancer specimens compared to normal epithelia, suggesting that SNEP5 expression is associated with differentiation of OSCC (Ding et al. 2008). On the other hand, Katayama et al. (2007) found that SUMO-1 is overexpressed in human OSCC cell lines and OSCC tissues from patients, and might be connected with tumor cell proliferation.

## Non-coding RNAs

One of the epigenetic mechanisms is regulation of non-coding RNAs (ncRNAs), which play important role in cellular homeostasis, development, and differentiation, as well as it may cause disease development, including cancer (Wang and Chang 2011; Esteller and Pandolfi 2017). ncRNAs are not translated into proteins and may be divided into classes based on their transcript size: small ncRNAs (including miRNAs, siRNAs, and piRNAs) and long ncRNAs (lncRNAs) such as long intergenic ncRNAs, circular RNAs, and pseudogene transcripts (Wang and Chang 2011; Osorio and Castillo 2016; Esteller and Pandolfi 2017; Wei et al. 2017). Below we will present some miRNAs and lncRNAs associated with HNSCC.

### miRNAs

MicroRNAs (miRNAs), one of the classes of small non-coding RNA, are short (17–25 nucleotides) single-stranded RNAs that are partially complementary to the 3′-untranslated region of messenger RNAs. Through binding to mRNAs they cause their degradation or inhibit their translation, and as a result they modulate expression of nearly 30% of human genes (Lee and Dutta 2009; Shiiba et al. 2010; Osorio and Castillo 2016). miRNAs participate in many cellular processes like proliferation, differentiation, development, and apoptosis (Bartel 2004; Kimura et al. 2010; Osorio and Castillo 2016). Furthermore, miRNAs may be classified as oncogenes or suppressor genes based on their cancer-related expression. A subgroup of miRNAs, epi-miRNAs, is associated with epigenetic factors like HDACs or DNMTs, suggesting that they may affect members of the epigenetic machinery and in consequence may influence gene expression. The level of miRNA expression is deregulated in cancer initiation and progression (Hatziapostolou and Iliopoulos 2011; Castilho et al. 2017) and recent studies find that miRNAs may be used as biomarkers for cancers (Shiiba et al. 2010; Irani 2016). Recent results concerning associations between miRNAs and HNSCC are presented below.

#### Oncogenic miRNAs

One of the best-known oncogenic miRNAs is miR-21, which has many targets genes of which most are suppressor genes like *PTEN*, *TPM1*, *TIMP3*, and *PDCD4* (Li et al. 2009; Selcuklu et al. 2009; Scapoli et al. 2010), suggesting that miR-21 plays a role in cancer invasion and metastasis (Zhu et al. 2008). In HNSCC, most of the target genes for miR-21 are tumor suppressors (Chen et al. 2013a), and meta-analysis shows that in HNSCC miR-21 is upregulated (Chen et al. 2012; Kumarasamy et al. 2019). Overexpression of miR-21 is associated with decreased 5-year survival in HNSCC patients (Avissar et al. 2009b). Li et al. (2009) revealed that overexpression of miR-21 is negatively correlated with expression of *PTEN* and *TPM1*, as well as associated with advanced clinical stage of TSCC, LN metastasis, and poor differentiation, suggesting that miR-21 may be a useful prognostic marker for patients with tongue cancer. Singh et al. (2018) observed that expression of mir-21 is significantly positively correlated with clinical stages I-IV of oral cancer.

miR-155-5p is another well-known oncogenic miRNA. In OSCC patients with metastasis to neck lymph nodes this miRNA is overexpressed, and is therefore suggested to be a poor prognostic factor, but also may be used as a novel target in oral cancer therapy (Baba et al. 2016). Overexpression of miR-155-5p was associated with TMN stage, LN metastasis, and poor differentiation also in LSCC (Cui et al. 2019). On the other hand, Rather et al. (2013) found that miR-155 targets *CDC73*, which is a tumor suppressor gene. In OSCC patients, overexpression of miR-155 decreases expression of *CDC73* and in consequence promotes cell proliferation as well as inhibiting apoptosis. It has been proposed that miR-155 plays an important role in regulation of cell growth through its target genes *CDC73* (Rather et al. 2013) and SOX10 (Cui et al. 2019), although it is upregulated in nasopharyngeal cancer (Chen et al. 2009).

miR-93 is overexpressed in HNSCC tissues and cell lines. Overexpression is associated with clinical stage, tumor progression, and LN metastasis, as well as inversely correlated with poor overall survival, suggesting that miR-93 might be an important factor in the progression of head and neck cancer (Li et al. 2015).

miR-211 has been recognized as targeting transforming growth factor-β type II receptor (*TGFβRII*) and thus promoting tumor progression. In HNSCC samples with metastasis, the expression of miR-211 is negatively correlated with expression of TGFβR2 protein, and in consequence is associated with poor prognosis for HNSCC patients (Chu et al. 2013). Zheng et al. (2018) also reported overexpression of miR-211 in tissues and cell lines from oral cancer, and interestingly found that higher miRNA expression is correlated with decreased expression of the tumor suppressor gene *BIN1* (bridging integrator-1) which may be a target of miR-211. Overexpression of BIN1 protein in OSCC cell lines is associated with decreased proliferation and migration, suggesting that miR-211 may be a new target in treatment of oral cancer.

Overexpression of miR-134 occurs in HNSCC patients, and high expression is connected with nodal metastasis and poor survival (Liu et al. 2014). Although miR-134 is upregulated in OSCC cell lines, its potential target gene *PDCD7* has a lower expression, an effect enhancing OSCC progression (Peng et al. 2018).

miR-205-5p is overexpressed in tumoral and peritumoral HNSCC tissues, and targets *RAD17* and *BRCA1*, DNA repair genes. Lower expression of *RAD17* and *BRCA1* may increase defects in DNA damage response and cause chromosomal instability (Valenti et al. 2019).

miR-31 is another oncogenic miRNA for HNSCC and is overexpressed in tissues and serum from HNSCC patients; furthermore an increased level is associated with TNM status and node stage. In addition, upregulation of miR-31 is connected with poor prognosis for HNSCC patients, suggesting its use as a prognostic marker (Wang et al. 2018a). miR-31 down-regulates the tumor suppressor gene *ARID1A* (AT-rich interacting domain) and decreases expression of ARID1A protein, a member of the chromatin remodeling SWI/SNF complex, and may inhibit stemness and oncogenicity. HNSCC patients with increased miR-31 and decreased ARID1A expression have poor survival (Lu et al. 2016). However, miR-31 is downregulated in laryngeal cancer cases and a low level of expression is associated with an advanced stage of cancer (Yang et al. 2018).

#### Tumor Suppressor miRNAs

Recent studies show that HNSCC patients have a low expression of miR-9. Moreover, in HNSCC cell lines, knockdown of miR-9 causes an increased invasiveness, cell cycle progression, cellular proliferation, and colony formation, and targets the gene *CXCR4*, a discovery which may be useful in therapy (Hersi et al. 2018). In NPC patients mir-9-3p is down-regulated, while its targets genes fibronectin 1 (*FN1*), β1 integrin (*ITGB1*), and α5 integrin (*ITGAV*) are upregulated. On the other hand, in NPC cell lines, higher expression of miR-9-3p decreases the proliferation, invasion, and migration of nasopharyngeal cancer cells (Ding et al. 2017).

Downregulation of miR-16 expression is observed in OSCC patients and cancer cell lines and its lower expression is negatively correlated with overexpression of its target gene Tousled-like kinase 1 (*TLK1*) and associated with positive LN metastasis, as well as with higher TNM stage and poor prognosis (Hu et al. 2018a). Other targets genes for miR-16, the oncogenes *AKT3* and *BCL2L2*, may promote cell proliferation and inhibit apoptosis in OSCC cells; oral cancer cell lines show a negative correlation between expression of miR-16 and expression of *AKT3* and *BCL2L2*, confirming a tumor-suppressing role of miR-16 in oral cancer (Wang and Li 2018).

A bioinformatics-based study showed that miR-99a-5p is downregulated in HNSCC and is negatively associated with expression of *PIK3CD* (phosphatidylinositol-4,5-bisphosphate 3-kinase catalytic subunit delta), which takes part in the PI3K-Akt signaling pathway, suggesting that it may be a tumor suppressor in head and neck cancer (Chen et al. 2018). miR-99a is also down-regulated in oral cancer and cell lines, but lower expression is not connected with the clinical stage; moreover, decreased expression of miR-99a promotes migration, proliferation, and cell invasion which are connected with higher expression of the *MTMR3* gene, a miR-99a target (Kuo et al. 2014). Yan et al. (2012) confirmed decreased expression of miR-99a in patients with oral cancer, and studies of TSCC cell lines show that higher expression of miR-99a inhibits cell growth and starts apoptosis. The *mTOR* gene (mammalian target of rapamycin), a serine/threonine protein kinase which plays an important role in regulating many pathways such as cell growth, cell survival, and differentiation is a potential target of miR- 99a (Watanabe et al. 2011; Yan et al. 2012). Wei et al. (2019) also reported reduced expression of miR99a-3p among HNSCC patients.

miR-34a is downregulated in HNSCC and reduced expression is characteristic for samples with LN metastasis; its target gene *AREG* (ligand of epidermal growth factor) takes part in tumor development, suggesting that miR-34a may play a key role in the suppression of invasion and metastasis in HNSCC (Zhang et al. 2015a). Other studies also observed downregulation of miR-34a (Scapoli et al. 2010; Kumar et al. 2012), and miR-34a expression is low in HNSCC cell lines (Kumar et al. 2012). Ectopic expression of miR-34a using in vitro and in vivo models caused inhibition of cell migration and proliferation of HNSCC cell lines. Moreover, miR-34a regulates tumor angiogenesis in head and neck cancer (Kumar et al. 2012).

Downregulation of miR-638 occurs in OSCC and is negatively correlated with TMN stages and LN metastasis. Besides, Tang et al. (2019) reported that restored expression of miR-638 inhibited migration, invasion, and proliferation of OSCC cells, and suggested that miR-638 might be a tumor suppressor by miR-638/wnt/ b-catenin axis.

miR-375 is downregulated nearly 22-fold in HNSCC tissues compared to normal tissues (Avissar et al. 2009a), suggesting that it may play a role in the transcriptional repression of an oncogene. Additionally, the expression ratio of miR-221 to miR-375 may serve as a cancer prognostic tool due to its high specific and sensitivity.

miR-625 may be a tumor suppressor miRNAs, because its level is lower in laryngeal squamous cell carcinoma (LSCC) and low expression is correlated with an advanced clinical stage of cancer and LN metastasis (Li et al. 2019b). On the other hand, miR-625 could be used in therapy because its overexpression decreases the invasion, proliferation, and migration of LSCC cells by targeting the gene *SOX4*.

Other studies and meta-analyses show that in HNSCC patients some miRNAs are upregulated, such as miR-126 and miR-223 in OSCC (Tachibana et al. 2016), miR-196b (Luo et al. 2019), miR-31 (Kao et al. 2019), miR-1275 in HNSCC (Liu et al. 2018), miR-212 and miR-129 in OSCC (Scapoli et al. 2010), and miR-130b in HNSCC (Chen et al. 2012), while others are down-regulated, such as miR-145-5p in LSCC (Gao et al. 2019), miR-29a in OSCC (Huang et al. 2019), miR-200b in HNSCC (Kumarasamy et al. 2019), miR-125a-5p in HNSCC (Vo et al. 2019), miR-486-3p and miR-337-3p in OSCC (Chou et al. 2019), miR-224 in OSCC (Lu et al. 2019), miR-135b, miR-197, miR-378, miR224, and miR-34a in OSCC (Scapoli et al. 2010), miR-100 and miR-375 in HNSCC (Chen et al. 2012).

### lncRNAs

lncRNAs are transcribed RNA molecules, which have a length of more than 200 nucleotides, do not encode proteins, and participate in positive and negative regulation of gene expression in the transcriptional, as well as the post-transcriptional level (Wang and Chang 2011; Chen 2016; Wei et al. 2017). Moreover, they regulate the transcription via modulation of chromatin structure and in consequence, are pivotal regulators of diverse biological processes, such as apoptosis, cell proliferation, metabolism, cell cycle, etc. (Akhade et al. 2017). lncRNAs may contribute to cancer development as oncogenes or tumor suppressors (Momen-Heravi and Bala 2018), thus might be used as biomarkers in diagnostics and target in therapy (Yang and Deng 2014).

An example of lncRNA associated with HNSCC development is HOXA11 antisense RNA (HOXA11-AS), which was found to be overexpressed in LSCC (Qu et al. 2018) and OSCC (Li et al. 2019a; Wang et al. 2019b). Upregulation of HOXA11-AS is significantly associated with poor prognosis of LSCC patients, while downregulation in LSCC cell lines is connected with inhibition of the invasion and migration of cancer cells, suggesting an oncogenic role of HOXA11-AS (Qu et al. 2018). Similarly, Li et al. (2019a) observed that higher expression detected in OSCC patients was correlated with lymph node metastasis, grade and clinical stage of oral cancer, while in OSCC cells in vitro it promoted proliferation. Moreover, bioinformatic analysis suggested that miR-518a-3p may be a target of HOXA11-AS, and in consequence a promoter of PDK1 expression in OSCC (Li et al. 2019a). On the other hand, Wang et al. (2019b) revealed another target of HOXA11-AS, miR-214-3p, which negatively regulated the proto-oncogene PIM1. Importantly, HOXA11-AS/miR-214-3p/PIM1 axis may be a potential target for oral cancer chemotherapy improvement. lncRNA *RHPN1-AS1* acts as an oncogene, which was confirmed by Qiu et al. (2019), who showed that *RHPN1-AS1* had a higher expression in HNSCC patients. Its knockdown was associated with significant inhibition of migration and invasion of HNSCC cell lines. Moreover, downregulation of *RHPN1-AS1* promoted apoptosis of cancer cells. In oral cancer, Guo et al. (2018) reported that lncRNA CEBPA-AS1 was upregulated in OSCC tissues and Tca8113 and Cal27 cell lines, suggesting that lncRNA CEBPA-AS1 may promote OSCC development. Moreover, the increased expression correlated with lymph node metastasis, poor differentiation, and high clinical stage of OSCC. The results indicated that lncRNA CEBPA-AS1 might be a novel prognostic biomarker and therapeutic target for patients with oral cancer. The lncRNA ST7-AS1 is the antisense transcript for ST7 (suppressor of tumorigenicity 7 protein) and plays an oncogenic role in LSCC. The ST7-AS1 overexpression in LSCC tissues and cell lines is associated with poor overall survival of LSCC patients. Qin et al. (2019) showed that interacting partner for ST7-AS1 was CARM1, which promoted metastasis and cancer development throughout its methyltransferase activity. In results, they showed a novel ST7-AS1/CARM1/Sox-2 signaling axis occurring in LSCC progression. lncRNAs may also inhibit HNSCC development, such as lncRNA LINC01133, which is downregulated in OSCC. However, the increased expression is associated with decreased metastasis and longer survival of OSCC patients, suggesting that LINC01133 may play a role of tumor suppressor gene (Kong et al. 2018). Downregulation of lncRNA AC026166.2-001 occurs in LSCC patients. Shen et al. (2018) reported that higher expression of AC026166.2-001 suppressed cell proliferation and migration in LSCC cells, inhibited cells cycle, and supported cell apoptosis in laryngeal cancer. Also other lncRNAs are upregulated in HNSCC and may display oncogenic properties, such as ZFAS1 (Kolenda et al. 2019), PVT1 (Yu et al. 2018), CASC9 (Sassenberg et al. 2019), TUG1 (Zhang et al. 2018b), MIAT (Zhong et al. 2019), SNHG20 (Wu et al. 2019), or RGMB-AS (Xu and Xi 2019), or downregulated and may act as tumor suppressor genes, such as STR5-AS (Wang et al. 2019a), C5orf66-AS1 (Lu et al. 2018), AC012456.4 (Hu et al. 2018b), LINC01133 (Kong et al. 2018), ZNF667, and ZNF667-AS1 (Meng et al. 2019).

## Chromatin Remodeling

Chromatin remodeling describes the dynamic changes of chromatin organization which influence regulation of gene transcription, replication of DNA, apoptosis, DNA repair, and also chromosome condensation and segregation (Wang et al. 2007a). Chromatin remodeling is undertaken by mechanism such as covalent histone modifications and DNA methylation which were described above, and also uses histone variants and ATP-dependent complexes of chromatin remodeling enzymes. Deregulation of chromatin remodeling may contribute to many diseases, including cancer (Wang et al. 2007b). ATP-dependent enzymes engaged in chromatin remodeling play important roles in regulation of gene transcription by modifying the organization of nucleosomes (Hatziapostolou and Iliopoulos 2011). Importantly, to remodel nucleosome organization, these ATPases utilize the energy from ATP hydrolysis. Chromatin remodeling ATPases are composed of four families, SWI/SNF, ISWI, NuRD/Mi-2/CHD, and INO80 (Bao and Shen 2007; Wang et al. 2007b). The SWI/SNF (switching/sucrose nonfermenting) family consists of two subfamilies, PBAF (polybromo-associated factor) and BAF (BRG1 or BRM-associated factor) (Halliday et al. 2009). SWI/SNF is essential in regulation of transcription, repair, recombination, and cell cycle progression as well as in the immune pathway and organ development, and in consequence nonfunctional complexes may influence carcinogenesis (Bao and Shen 2007; Halliday et al. 2009). The SWI/SNF complex also contains bromodomain units (Halliday et al. 2009). BRD7 (bromodomain-containing protein 7), a subunit of the PBAF complex, is hypermethylated in 74% of OSCC cases examined (Balasubramanian et al. 2015). Immunoreactivity of BAF250a, another subunit of SWI/SNF, is reduced to various levels in invasive OSCC cells compared to normal oral epithelial cells and is connected with poor outcome in OSCC patients with early pathological T-stage (T1/T2) without lymph node metastasis. However, no association is found between lower BAF250a immunoreactivity and smoking or alcohol abuse, gender, age, or LN metastasis (Inoue et al. 2018).

RSF1 is one subunit of ISWI remodeling factors, and its expression is upregulated in OSCC. Moreover, higher expression is correlated with poor overall survival in patients with oral cancer and is also associated with LN metastasis, as well as with advanced clinical stage of tumor and recurrent disease (Fang et al. 2011).

The Mi-2/NuRD (Nucleosome Remodeling Deacetylase) complex is also an important complex for chromatin remodeling. One of its subunits is DOC1 (Deleted in Oral Cancer 1) (Bao and Shen 2007; Wang et al. 2007b) whose loss is connected with OSCC; in OSCC cell lines, re-expression of DOC1 decreases cell proliferation or migration and induces a mesenchymal–epithelial transition (Mohd-Sarip et al. 2017).

The chromatin remodeling complex INO80 is also required for correct regulation of transcription and organization of nucleosomes, and incorrect function of its subunits may influence carcinogenesis (Bao and Shen 2007; Wang et al. 2007b).

## Conclusions

Recent studies show clearly that epigenetic mechanisms play important roles in head and neck carcinogenesis. Aberrant methylation of repeat sequences like LINE1 or tumor suppressors such as *DAPK, RASSF1* and*, ECAD* is undoubtedly crucial in tumor progression. Moreover, epigenetic alteration connected with histone modification and chromatin remodeling may cause open chromatin structure and facilitate transcriptions of factors involved in human malignancies. Also expression of microRNAs may influence tumor progression and in consequence the prognosis for patients. On the other hand, knowledge about dysregulated microRNAs and their target genes may improve therapeutic strategies. Importantly, utilizing information about hypo- or hypermethylation markers may be useful and reliable for early detection and prognosis. Because epigenetic changes are reversible, further research about aberrant patterns of epigenetic events is important to provide better and more effective therapies for patients with head and neck cancer.
